# Comparative seismic performance of steel EBF shear links frame designed to IS 18168:2023 using force-based and direct displacement method

**DOI:** 10.1038/s41598-026-47433-6

**Published:** 2026-04-11

**Authors:** Bush Rc, Venkata Vamsi Emani, Ahmad Batah, Mohamed F. Suleiman, Rajneesh Sharma, Varsha Rani, Rohit Vyas, Abdullah Ansari, Pranjal Mandhaniya, Ayed E. Alluqmani, Anoop I. Shirkol

**Affiliations:** 1 Department of Civil Engineering, Malaviya National Institute of Technology, 302017 Jaipur, Rajasthan India; 2 AR-TEC Engineering & Innovations, Roermond, Netherlands; 3 Aguirre Project Resources LLC, 75068 Texas, USA; 4 Department of Infrastructure Engineering, The University of Melbourne, Parkville, VIC 3052 USA; 5 Civil Engineering Department, University of Tripoli, Tripoli, Libya; 6 Dept. Of Civil Engineering), Engineering College Jhalawar, Jhalawar, India; 7 Government Polytechnic, Purnea, Bihar India; 8 Earthquake Monitoring Center, Sultan Qaboos University, Muscat, Al Khoudh, PC: 123 Oman; 9 Department of Mechanical and Industrial Engineering, Norwegian University of Science and Technology Trondheim, Trondheim, Norway; 10 Department of Civil Engineering, Faculty of Engineering, Islamic University of Madinah, Al-Madinah Al-Munawarah, Medina, Saudi Arabia

**Keywords:** Eccentrically Braced Frames (EBFs), Direct Displacement-Based Design (DDBD), Seismic Performance Evaluation, Nonlinear Dynamic Analysis, IS 18168:2023, Engineering, Mathematics and computing

## Abstract

The recent introduction of IS 18168:2023 marks a significant advancement in the seismic design of steel structures in India by providing dedicated provisions for eccentrically braced frames (EBFs). While the code promotes link-controlled energy dissipation, its performance under different design philosophies, particularly in the nonlinear range, remains largely unexplored. This study presents a comprehensive seismic performance evaluation of steel EBF buildings designed as per IS 18168:2023 using conventional Force-Based Design (FBD) and Direct Displacement-Based Design (DDBD) approaches. The nonlinear static pushover analysis, nonlinear time-history analysis, and incremental dynamic analysis are performed for four building heights of 3-, 6-, 9-, and 12-storey. In addition, various important response parameters, including inter-storey drift, link rotation, plastic hinge distribution, variability, and collapse-related behaviour, have been systematically examined. Based on the results, it has been confirmed that EBF systems designed according to IS 18,168 exhibit a stable and desirable seismic behaviour, with inelastic deformation pretty much localized in the shear links. With respect to FBD, however, DDBD provides stronger control over nonlinear response due to approximately 20–40% lower median drift demands, 30–50% reduction at critical link rotation in MCE level, and strongly reduced record-to-record variability. Further, the drift escalation in DDBD frames is delayed while collapse robustness is notably improved, particularly for the midand high-rise buildings. Overall, the study brings out that though IS 18,168 provides a robust codal framework in the design of EBF systems, integrating DDBD within this codal context further leads to a more realistic, predictable, and performanceoriented seismic response, especially under strong ground motions.

## Introduction

**Fig. 1:**
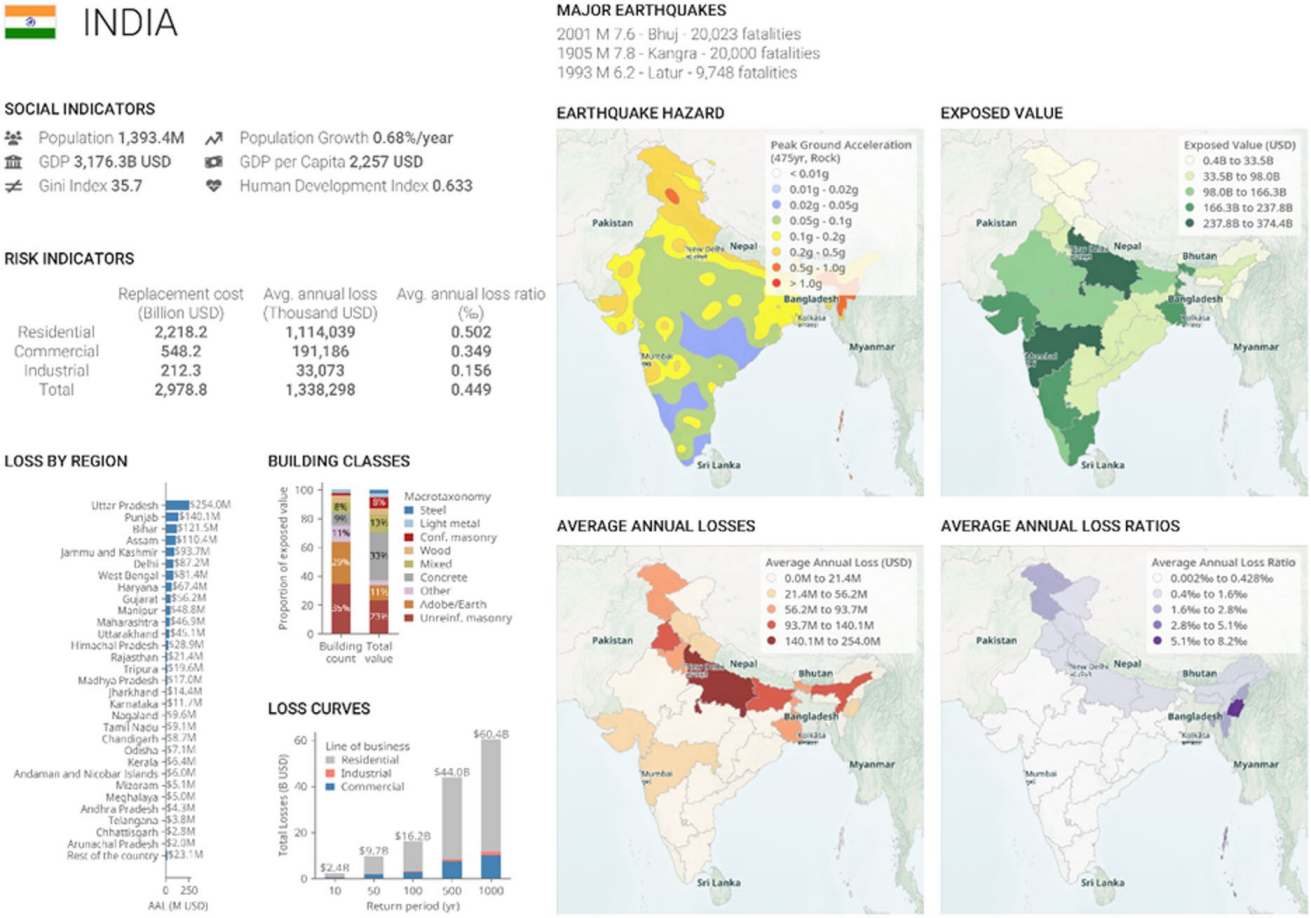
Seismic hazard and risk distribution of India highlighting major earthquake zones, exposed values, and average annual losses. [adapted from GEM Foundation, India Seismic Risk Profiles, 2023, CC BY-NC-SA 4.0 (https://www.globalquakemodel.org/product/seismic-risk-profiles)]^40^

India is highly vulnerable to seismic hazards, with nearly 18–20% of its territory located in the high seismic hazard regions classified as Seismic Zones IV and V (Fig. 1)^1^. Several devastating earthquakes, such as the 1905 Kangra (Mw 7.8), 1993 Latur (Mw 6.2), and 2001 Bhuj (Mw 7.6) events, have repeatedly demonstrated the nation’s exposure to strong ground shaking and widespread structural damage^2^. National loss estimates further indicate that the average annual loss (AAL) in the residential and commercial building sectors exceeds USD 1.3 billion, underscoring the urgent need for robust and resilient seismic design strategies^3^. While reinforced concrete buildings dominate Indian construction practice^4^, steel structures offer significant advantages in seismic regions, including high strength-to-weight ratio, superior ductility, and predictable inelastic behaviour, making them particularly suitable for performance-based seismic design^5^.

In this context, the recently published Indian Standard IS 18,168^6^ represents an important milestone by introducing dedicated provisions for the seismic design of steel special moment resisting frames and eccentrically braced frames (EBFs) in moderate to severe seismic zones. Among various steel lateral load-resisting systems, EBFs combine the high stiffness of concentrically braced frames with the superior energy dissipation capacity of moment resisting frames, achieved through the controlled yielding of short link beams^7^. This hybrid stiffness–dissipation mechanism enables EBFs to limit excessive drift while ensuring stable hysteretic energy dissipation during strong ground shaking^8^. Common EBF configurations, including diagonal, chevron (Inverted-V), and V-type arrangements, are defined in IS 18,168^6^. The inclusion of EBFs in IS 18168:2023 marks a significant step toward enhancing the seismic resilience of Indian steel buildings.

Despite these advancements, current Indian seismic design practice remains largely governed by force-based design (FBD) procedures, as prescribed in IS 1893:2016^1^. Force-based approaches rely on elastic analysis combined with empirical response reduction factors to indirectly account for inelastic behavior^9^. Although computationally convenient, such methods do not explicitly control displacement or deformation demands and often fail to capture realistic nonlinear response characteristics, particularly in mid- and high-rise steel frames where brace axial deformation and link beam rotation govern global behavior^10^. As a result, FBD-designed structures may exhibit unintended concentration of inelasticity, excessive inter-storey drifts, and uncertain performance under strong earthquakes^11^.

In contrast, Direct Displacement-Based Design (DDBD) has emerged internationally as a rational performance-based seismic design methodology in which target displacement and deformation limits are explicitly defined at the outset of the design process^10,12–16^. By directly linking seismic demand to displacement capacity, DDBD enables more reliable control of damage, ductility, and energy dissipation mechanisms. Several studies have demonstrated the effectiveness of DDBD for reinforced concrete and steel structural systems^16–18^; Sullivan (2013)^13^ introduced a DDBD framework for steel EBF structures and demonstrated, through nonlinear time-history analyses, that displacement-based design can overcome key limitations of conventional force-based approaches in controlling global displacements and storey drifts. Subsequently, O’Reilly and Sullivan (2015)^15^ further refined this methodology by calibrating spectral displacement reduction factors using experimentally validated numerical models and verified its effectiveness for EBFs ranging from low- to high-rise configurations, with particular emphasis on systems with short links. In parallel, Bosco, Marino, and Rossi (2015)^19^ critically reviewed the Eurocode 8 (EC8)^20^ design provisions for eccentrically braced frames and highlighted fundamental shortcomings in force-based procedures related to link over strength evaluation, treatment of P–Δ effects, and capacity design rules. More recently, Kalapodis et al. (2022)^21^ extended the DDBD framework by adopting an equivalent MDOF representation and demonstrated improved drift control and design efficiency for EBF structures when compared with Eurocode-8-based force design^20^, while also referring to force-based seismic design provisions adopted in international codes such as ASCE 7-22^22^ and NZS 1170.5^23^. Collectively, these studies demonstrate that while the seismic performance of EBFs and displacement-based design concepts have been extensively explored under international codes such as EC8^20^, ASCE 7-22^22^, and NZS 1170.5^23^.

however, its application to eccentrically braced steel frames within the Indian seismic context remains limited, particularly in conjunction with the newly introduced IS 18,168 2023 provisions. Notably, IS 18168:2023^6^ primarily follows force-based design principles and does not explicitly provide a displacement-based framework for performance evaluation of EBF systems, highlighting a critical research gap.

In order to fill this research gap, this research focuses on exploring the viability and effectiveness of Direct Displacement-Based Design for steel EBFs designed as per IS 18168:2023, and compares it with the force-based design approach to evaluate and compare the performance of steel EBFs designed using these two approaches. A rigorous analysis procedure involving nonlinear static pushover analysis (NSPA) and nonlinear time history analysis (NLTHA) using spectrum-compatible ground motions that simulate extreme seismic design conditions is also carried out. Additionally, this research also adopts Incremental Dynamic Analysis (IDA) to effectively represent the nonlinear behavior of EBF systems subjected to different levels of increasing seismic intensity. Following this, using these analysis methodologies, various performance parameters such as inter-storey drift, link rotation, base shear, and collapse risk are also estimated. The results derived from this research are hoped to help fill this research gap and provide quantitative information on the efficacy and limitation of DDBD for steel EBFs designed as per IS 18168:2023^6^.

## Methodology

### Design approaches adopted in the study

In this study, the seismic performance of steel EBF buildings is evaluated using both DDBD and conventional FBD approaches, in accordance with the relevant Indian seismic design provisions. The use of these two design methodologies enables a direct comparison between displacement-controlled and force-controlled seismic design philosophies for EBF systems.

**Fig. 2:**
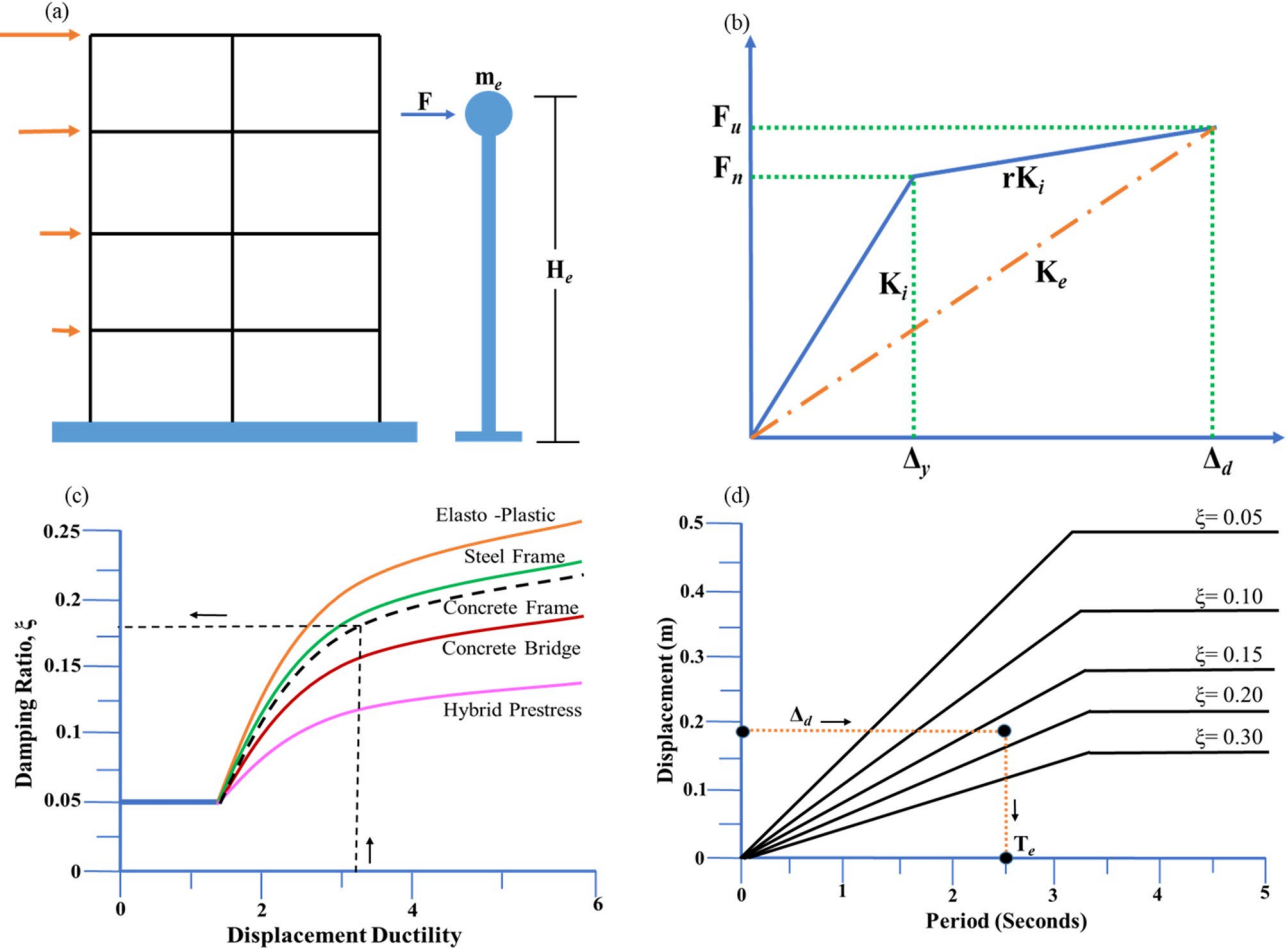
Fundamentals of the Direct Displacement-Based Design (DDBD) approach: (**a**) equivalent SDOF idealization; (**b**) effective secant stiffness; (**c**) damping–ductility relationship for various systems; and (**d**) displacement spectra for target displacement estimation.

The DDBD approach is based on the principle that seismic performance is more rationally governed by explicitly controlling displacement demand rather than forces. Accordingly, the multi-degree-of-freedom (MDOF) structural system is represented by an equivalent single-degree-of-freedom (SDOF) system, as shown in Fig. 2**(a)**^10^, which captures the dominant dynamic response of the structure through an effective mass and an effective height. This representation allows the global seismic response to be expressed in terms of a target displacement corresponding to the desired performance level.

Unlike force-based design, which relies on initial elastic stiffness, DDBD employs an effective secant stiffness defined at the target displacement level. This concept is schematically illustrated in Fig. 2**(b)**^13^, where the stiffness is evaluated from the force–displacement relationship at maximum displacement demand, enabling a realistic representation of the inelastic behaviour of the structure.

The energy dissipation associated with inelastic deformation is incorporated in the DDBD framework through equivalent viscous damping. The conceptual relationship between displacement ductility and equivalent viscous damping for different structural systems is illustrated in Fig. 2**(c)**. As ductility demand increases, the effective damping of the system also increases, reflecting enhanced hysteretic energy dissipation, which is particularly relevant for steel EBFs with stable yielding mechanisms.

The influence of damping on seismic displacement demand is accounted for using damping-modified displacement response spectra, as shown in Fig. 2**(d)**. The target design displacement is obtained from the intersection of the effective period of the substitute SDOF system with the appropriate damping-adjusted displacement spectrum. This displacement governs the subsequent evaluation of effective stiffness, base shear, and force distribution in the structure, forming the basis of the DDBD procedure.

For comparison purposes, the same EBF buildings are also designed using the conventional Force-Based Design approach in accordance with IS 1893:2016^1^. In this method, seismic demand is quantified in terms of equivalent lateral forces derived from the seismic weight, fundamental period, and codal design spectrum. The equivalent static lateral force method is employed, and the resulting base shear is distributed along the building height as per code provisions. Structural members are analyzed and designed following IS 18,168 2023 and IS 800:2007^24^, ensuring appropriate strength hierarchy among beams, columns, braces, and link elements.

While the FBD approach remains widely used in engineering practice due to its simplicity and direct code compliance, it does not explicitly control displacement demand or deformation-based performance. These limitations are particularly significant for eccentrically braced frames, where seismic response is governed by brace axial deformation and link rotation. This motivates the comparison between FBD and DDBD in the present study, especially within the context of Indian seismic design practice. The key input parameters and base shear calculations for the representative 6-storey frame are summarized in Table 1, and similar procedures are followed for the remaining building configurations.

**Table 1:** Seismic Base Shear and Lateral Load Distribution as per IS 1893:2016.

Parameter	Description	Value
Design Parameters	Seismic Zone (Z)	0.36 (Zone V)
	Importance Factor (I)	1.2 (Public Building)
	Response Reduction Factor (R)	5.0
	Soil Type	Medium
	Damping Ratio	5%
Fundamental Time Period	As per IS 1893:2016, Cl. 7.6.1: T = 0.075 h^0.75	T = 0.075(21)^0.75 = 0.735 s
Spectral Acceleration (Sa/g)	For clay soil and T = 0.735 s:	Sa/g = 2.27
Design Horizontal Acceleration Coefficient	Ah = (Z/2) × (Sa/g) ÷ (R/I)	Ah = 0.0981
Design Seismic Weight (W)	Total seismic weight of the building	12007.44 kN
Design Base Shear	V_B_ = Ah × W	V_B_ = 0.0981 × 12007.4 = 1180 kN

### Analytical framework for DDBD

In the present study, short shear links are adopted in the displacement-based design of EBFs. The selected link section ISMB 400 with an assumed link length of 0.60 m satisfies the shear link criterion $\:{L}_{l}\le\:1.6{M}_{p}/{V}_{p}$. Shear links are preferred due to their stable hysteretic response, high energy dissipation capacity, and consistent performance under cyclic loading. Once the link type is confirmed, the storey yield drift ratio $\:{{\uptheta\:}}_{y}$, represents the deformation level at which yielding initiates in the frame, is evaluated by combining the contributions from brace elongation and link shear deformation. The brace elongation contribution is expressed using the yield strain of the brace ($\:{\epsilon}_{yb}$ of 0.001725) together with the geometric ratio $\:{L}_{bay}/{H}_{s}$, where bay length **(**$\:{L}_{bay}$) of 6.0 m and storey height ($\:{H}_{s}$) of 3.5 m, representing the axial elongation of the braces at yielding.

The second contribution accounts for shear deformation of the link, evaluated using ($\:0.55{h}_{l}{t}_{w}{\epsilon}_{yl}{L}_{l}^{3})/(12I{L}_{bay})$, where the link depth ($\:{h}_{l}$) is 400 mm, web thickness ($\:{t}_{w}$) is 8.9 mm, the, and the moment of inertia ($\:I$) is 2.02 × 10⁻⁴ m⁴. The link shear strength is calculated as $\:{V}_{p}=0.55{h}_{l}{t}_{w}{f}_{y}$ giving $\:{V}_{p}=\:675.51$ kN for the yield strength $\:{f}_{y}=345\:\mathrm{N}/\mathrm{m}\mathrm{m}^2$, The corresponding shear stiffness is obtained as $\:{K}_{s}=12EI/{L}_{l}^{2}$ is 1.346 × 10⁷ N/m, resulting in a shear displacement $\:{{\updelta\:}}_{s}={V}_{p}/{K}_{s}$ $\:=0.000502\:$m. Dividing $\:{{\updelta\:}}_{s}$ by *L*_*l*_ gives the yield rotation ($\:{{\upgamma\:}}_{l}=0.000836\:\mathrm{r}\mathrm{a}\mathrm{d}$), and including the brace elongation component yields a total storey yield drift ratio which, when multiplied by the link-to-bay length ratio, provides the drift angle ($\:{{\uptheta\:}}_{y}$) of 0.00304 rad, equivalent to 0.304% or 0.174**°**.

The total storey drift capacity is evaluated by combining the yield drift with the additional plastic rotation capacity of the link, expressed as $\:{{\uptheta\:}}_{c,\:i}={{\uptheta\:}}_{y,\:i}+{{\uptheta\:}}_{p,\:i}$, $\:{{\uptheta\:}}_{y,\:i}$is the yield drift ratio and $\:{{\uptheta\:}}_{p,\:i}={\mathrm{e}}_{i}\:\times\:{{\upgamma\:}}_{p,link,\:i\:}/{\mathrm{L}}_{b}$ Here, $\:{\mathrm{e}}_{i}\:$denoting the link length, $\:{{\upgamma\:}}_{p,link,\:i\:}$the plastic chord rotation capacity of the link, and $\:{\mathrm{L}}_{b}$ the bay length. performance limit states are defined based on experimental evidence, where the “no damage” state corresponds to the yield rotation $\:{{\upgamma\:}}_{y,\:}$​, the “repairable damage” state allows a small inelastic increment beyond yield ($\:{{\upgamma\:}}_{y,\:}+0.08$ for shear links and $\:{{\upgamma\:}}_{y}+0.02$ for flexural links)^13^, and the “no collapse” state permits the maximum plastic demand without structural failure ( $\:{{\upgamma\:}}_{y}+0.10$ for shear links and $\:{{\upgamma\:}}_{y}+0.025$ for flexural links). These values, recommended by Engelhardt and Popov (1989)^25^ and refined by Sullivan (2013)^13^, provide a rational basis for relating drift capacities to structural performance. The corresponding total storey drift capacities are summarized in Table 2.

**Table 2:** Total storey drift capacities of the EBF frame corresponding to different damage states.

Limit state	No damage	Repairable damage (radian)	No collapse (radian)
Limit state units (radian)	0.00304	0.0110	0.0130

Once the total storey drift capacity is defined, the displacement profile of the structure is established to distribute these limits along the height of the building. For storeys governed by the critical drift limit, the displacement at level *i* is given by $\:{{\Delta\:}}_{i}={{\uptheta\:}}_{c}{\mathrm{h}}_{i}$ where $\:{\mathrm{h}}_{i}$​ is the height of the level above the base. When the critical drift exceeds the yield drift ($\:{{\uptheta\:}}_{c}>{{\uptheta\:}}_{y}),$ the displacement profile accounts for both yield and plastic components and is expressed as $\:{{\Delta\:}}_{i}={{\uptheta\:}}_{y}{\mathrm{h}}_{i}+{{\uptheta\:}}_{c}-{{\uptheta\:}}_{y}){\mathrm{h}}_{i}\times\:\frac{{(2H}_{n}-{h}_{i})}{{(2H}_{n}-{h}_{1})}$, where $\:{H}_{n}$ is the total building height and $\:{h}_{1}$​ is the first-storey height. The Table 3**(Part a)** presents the resulting displacement profile along the height of the structure for different damage states, ensuring that inelastic deformation demand is realistically distributed with larger contributions at upper storeys.

The design displacement $\:{{\Delta\:}}_{d}$ is then defined to transform the multi-degree-of-freedom (MDOF) system into an equivalent single-degree-of-freedom (SDOF) representation. For a selected target performance level, the design displacement is calculated as $\:{{\Delta\:}}_{d}={\sum\:m}_{i}{{\Delta\:}}_{i}^{2}/\sum\:{m}_{i}{{\Delta\:}}_{i}$ where $\:{m}_{i}$ (204000 kg) and $\:{{\Delta\:}}_{i}$​ (0.633) for the repairable condition and 0.736 for no collapse condition. The effective modal properties of the equivalent SDOF system are obtained using $\:{M}_{effeective}=({\sum\:{m}_{i}{{\Delta\:}}_{i})}^{2}/\sum\:{m}_{i}{{\Delta\:}}_{i}^{2})$, $\:{H}_{effective}={\sum\:m}_{i}{{\Delta\:}}_{i}{\mathrm{H}}_{i}/\sum\:{m}_{i}{{\Delta\:}}_{i}$, The design displacement, effective mass, and effective height for both performance levels are summarized in Table 3**(Part b)**.

To ensure compatibility with standard response spectra, the design displacement is converted to an equivalent 5% damping level. The equivalent viscous damping is evaluated as $\:{{\upxi\:}}_{eq}=0.05+0.577({\upmu\:}-1/{\upmu\:}$​$\:{\uppi\:}$), where the ductility demand ratio, defined as $\:\:{\upmu\:}={\sum\:}_{i=1}^{n}{u}_{i}{V}_{i}{\theta\:}_{i}/{\sum\:}_{i=1}^{n}{\theta\:}_{i}$Here, $\:{u}_{i}={\theta\:}_{i}/{\theta\:}_{y,i}=({{\Delta\:}}_{i}-{{\Delta\:}}_{i-1})/\left[{\theta\:}_{y,i}\left({\mathrm{h}}_{i}-{\mathrm{h}}_{i-1}\right)\right]$. The spectral reduction factor$\:\:\:\:{\upeta\:}=({\frac{0.07}{0.02+{{\upxi\:}}_{eq}})}^{{a}_{SF}}$is applied, with $\:{a}_{SF}$ typically taken as 0.5 for far-field and 0.25 for near-field earthquakes, following Priestley et al. (2007)^10^. The equivalent 5% damped design displacement is then obtained as $\:{{\Delta\:}}_{d\:}({\mathrm{T}}_{e},5\mathrm{\%})={{\Delta\:}}_{d\:}\left({\mathrm{T}}_{e}\right)/{\upeta\:}$. All the parameters computed through the above formulations, including the ductility ratio, equivalent viscous damping, spectral reduction factor, and the corresponding 5% damped design displacements, are summarized in Table 3**(Part c)**.

**Table 3:** Storey-wise displacement and drift demands of the EBF frame along with global performance parameters at different limit states.

Part A: Storey-wise displacement and drift response								
Storey	Height (m)		Δ (Repairable)		Δ (No collapse)	θ_i_ (Repairable)		θ_i_ (No collapse)
1	3.5		0.038		0.045	0.011		0.013
2	7.0		0.072		0.084	0.009		0.011
3	10.5		0.100		0.117	0.008		0.0094
4	14.0		0.124		0.144	0.006		0.0070
5	17.5		0.142		0.164	0.005		0.0057
6	21.0		0.155		0.178	0.003		0.0039
**Part B: Global effective system parameters**								
**Performance state**		**Δ**_**d**_ **(m)**		**M**_**eff**_ **(kg)**			**H**_**eff**_ **(m)**	
Repairable		0.121		1067508.20			14.51	
No collapse		0.140		1072810.74			14.46	
**Part C: Performance and spectral parameters**								
**Parameter**				**Value**				
Ductility ratio, µ				2.398				
Equivalent viscous damping, ($\:{{\upxi\:}}_{eq}\mathrm{\%}$)​				15.7				
Spectral reduction factor, ($\:{\upeta\:}$)				0.629				
$\:{{\Delta\:}}_{d\:}$​(Te,5%) – Repairable (m)				0.192				
$\:{{\Delta\:}}_{d\:}$​ (Te,5%) – No collapse (m)				0.223				

The effective period $\:{T}_{e}$ is obtained by equating the target displacement $\:{{\Delta\:}}_{d\:}({\mathrm{T}}_{e},5\mathrm{\%})$ with the elastic spectral displacement. $\:{\mathrm{S}}_{d}={\Delta\:}\left(\mathrm{T}\right)=\frac{{\mathrm{S}}_{a}\times\:g{T}^{2}}{{4\pi\:}^{2}}$. The effective stiffness of the substitute SDOF system is then calculated as $\:{K}_{e}={4{\uppi\:}}^{2}\frac{{M}_{effective}}{{T}_{e}^{2}}$, and the corresponding design base shear is obtained as $\:{\mathrm{V}}_{b\:}={\mathrm{K}}_{e\:}\times\:{{\Delta\:}}_{d\:}+C\frac{{\sum\:}_{i=1}^{n}{P}_{i}{{\Delta\:}}_{i}}{{H}_{effective}}$, where the first term represents the elastic shear demand corresponding to the design displacement $\:{{\Delta\:}}_{d\:}$, and the second term accounts for additional effects due to gravity-induced second-order P–Δ moments. Here,$\:\:{P}_{i}$ is the seismic weight at storey $\:i$, $\:{{\Delta\:}}_{i\:}$is the lateral displacement at that level,$\:\:{H}_{effective}$​ is the effective height, and $\:C$ is a constant that equals 1.0 for steel structures when $\:{m}_{e}\times\:g{\times\:{\Delta\:}}_{d\:}/{\mathrm{V}}_{b\:}{\times\:H}_{effective}\ge\:0.05$, but otherwise reduces to zero as suggested by Priestley et al. (2007)^10^. The base shear is distributed along the height according to $\:{\mathrm{F}}_{i\:}=k\frac{{m}_{i}{{\Delta\:}}_{i}}{\sum\:{m}_{i}{{\Delta\:}}_{i}}{\mathrm{V}}_{b\:}$, while at the top storey, a slightly modified distribution is used, $\:{\mathrm{F}}_{i\:}=\left(1-k\right){\mathrm{V}}_{b\:}+k\frac{{m}_{i}{{\Delta\:}}_{i}}{\sum\:{m}_{i}{{\Delta\:}}_{i}}{\mathrm{V}}_{b\:}$ where $\:k$ is a scaling factor to balance higher-mode effects. For EBFs taller than six storeys, a value of $\:k$ =0.9 is recommended, whereas for shorter frames $\:k$ may be taken as 1.0. The storey shear is obtained as $\:{\mathrm{V}}_{i}=\sum\:_{j=i}^{n}{F}_{j}$ where $\:{F}_{j}$​ is the lateral force at storey $\:j$. and the corresponding link shear and moment demands are evaluated as $\:{V}_{link,i}=\frac{{V}_{i}{\mathrm{h}}_{s,\:i}}{{L}_{b}}$, where $\:{\mathrm{h}}_{s,\:i}$​ is the inter-storey height and $\:{L}_{b}$​ is the bay length, thereby translating storey shear into link shear demand. Furthermore, the plastic moment demand in the link is given by $\:{M}_{link,i}=\frac{{V}_{link,\:i}\times\:{\mathrm{e}}_{\:i}}{2}$ with $\:{\mathrm{e}}_{\:i}$representing the link length. This formulation reflects the equilibrium of forces and moments within each bay, directly linking the global shear demand to the local link design forces.

The excess-strength ratio is evaluated as the ratio of the resisting shear capacity of the link ($\:{V}_{R,\:link}/$) to its elastic shear demand $\:{(V}_{E,\:Link}$​) ($\:{V}_{y,\:link}={F}_{y}/{V}_{E,\:Link}$. The yield shear strength of the link $\:{V}_{y,\:link}$ is first calculated using the material yield strength $\:{F}_{y}$​, web thickness $\:{t}_{w}$, flange thickness $\:{t}_{f}$​, and section depth $\:h$. The resisting shear capacity depends on the ductility demand: for low ductility (µ < 0.1), it is obtained as ($\:{{\uptheta\:}}_{i}$/$\:{{\uptheta\:}}_{y,i}$)$\:\:{V}_{y,\:link,i}$, while for higher ductility levels (µ > 0.1), it is enhanced to account for additional plastic rotation demand, expressed as $\:{V}_{y,\:link,i}(1+0.5\frac{{V}_{p,i}}{{V}_{p,u}})$. The plastic rotation demand $\:{V}_{p,i}$​ is linked to the inelastic chord rotation ($\:{\theta\:}_{i}-{\theta\:}_{y,i})$ scaled by the bay length to link length ratio $\:({L}_{b}/{e}_{i})$ On the demand side, the elastic shear force $\:{V}_{E,\:Link}$ is computed from the story shear $\:{(V}_{i})$ and geometry parameters as $\:\left({V}_{i}{h}_{s,i}\right)/{L}_{b}$ By taking the ratio $\:{V}_{R,\:link}/{V}_{E,\:Link}$ the design checks whether the link has sufficient overstrength relative to elastic demand, thereby ensuring adequate ductility and compliance with Euro code 8 (1998)^28^ provisions. Table 4 summarises the displacement-based design parameters and the corresponding base shear for the 6-storey EBF frame. Compared with the FBD base shear of 1180 kN, the DDBD base shear is about 21% lower at the repairable limit state and 9% lower at the no-collapse state. This reduction occurs because DDBD links seismic demand to target displacement and effective stiffness, whereas FBD scales elastic spectral forces with the response-reduction factor R.

**Table 4:** Displacement-based design parameters and base shear for the 6-storey EBF frame.

Parameter	Unit	Repairable (Case A)	No-collapse (Case B)
Total mass, ∑m_i_	kg	1,224,000	1,224,000
Total weight, W = ∑m_i_ g	kN	12,007.44	12,007.44
Storey displacements Δ_i_ (1 to 6)	m	0.038, 0.072, 0.100, 0.124, 0.142, 0.155	0.045, 0.084, 0.117, 0.144, 0.164, 0.178
ΣΔ_i_	m	0.633	0.736
Σm_i_Δ_i_	kg·m	129,132.0	150,144.0
Design displacement Δd(Te,5%)	m	0.19249	0.22000
Effective mass m_eff_	kg	1,067,508.20	1,072,810.74
Effective height H_eff_	m	14.51	14.46
Global ductility µ	-	2.40	—
Equivalent viscous damping ξeq	-	0.15708	—
Spectral reduction factor η	-	0.6287	—
Effective stiffness K_e_	N/m	4.84 × 10⁶	4.84 × 10⁶
Design base shear V_b_	kN	932	1077

### Structural Configuration and Numerical Modelling

**Fig. 3:**
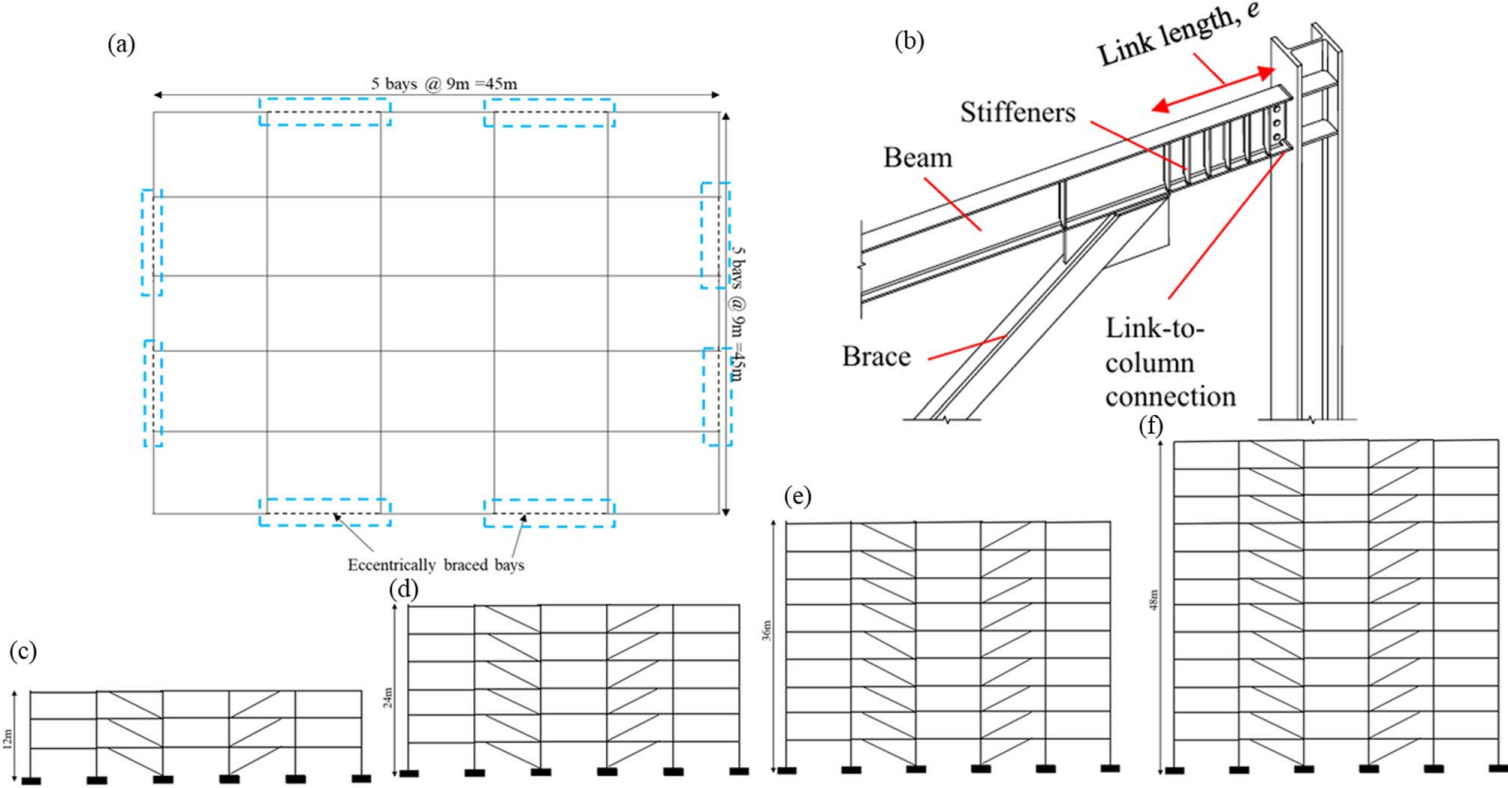
**(a-f).** Structural configuration and detailing of EBFs, showing plan layout, typical link beam–brace connection with stiffeners, and elevation views for different building heights.

In this study, four building configurations comprising 3-, 6-, 9-, and 12-storey eccentrically braced steel frames were analysed to investigate the influence of building height on seismic performance. The building plan is regular and symmetric in both principal directions, with uniform bay spacing and mass distribution. Accordingly, torsional effects were verified to be negligible, and the seismic response was dominated by translational modes. On this basis, a two-dimensional (2D) plane frame model representing the eccentrically braced bays was adopted for numerical analysis. This modelling approach is consistent with common practice for regular steel braced frames and enables clear identification of link-dominated inelastic mechanisms. Figure 3**(a-b)** presents the plan layout with five bays in each direction, while Fig. 3**(c–f)** show the elevation views of the 3-, 6-, 9-, and 12-storey frames considered in the study.

All models were developed in ETABS^26^, with beams, columns, braces, and link elements explicitly modelled using frame elements. Soil–structure interaction effects were neglected, and fixed-base boundary conditions were assumed for all frames to represent rigid foundation behaviour. Floor slabs were modelled as rigid diaphragms, considering the presence of relatively stiff floor systems and the absence of significant in-plane diaphragm deformation in steel buildings of the selected configurations.

**Fig. 4:**
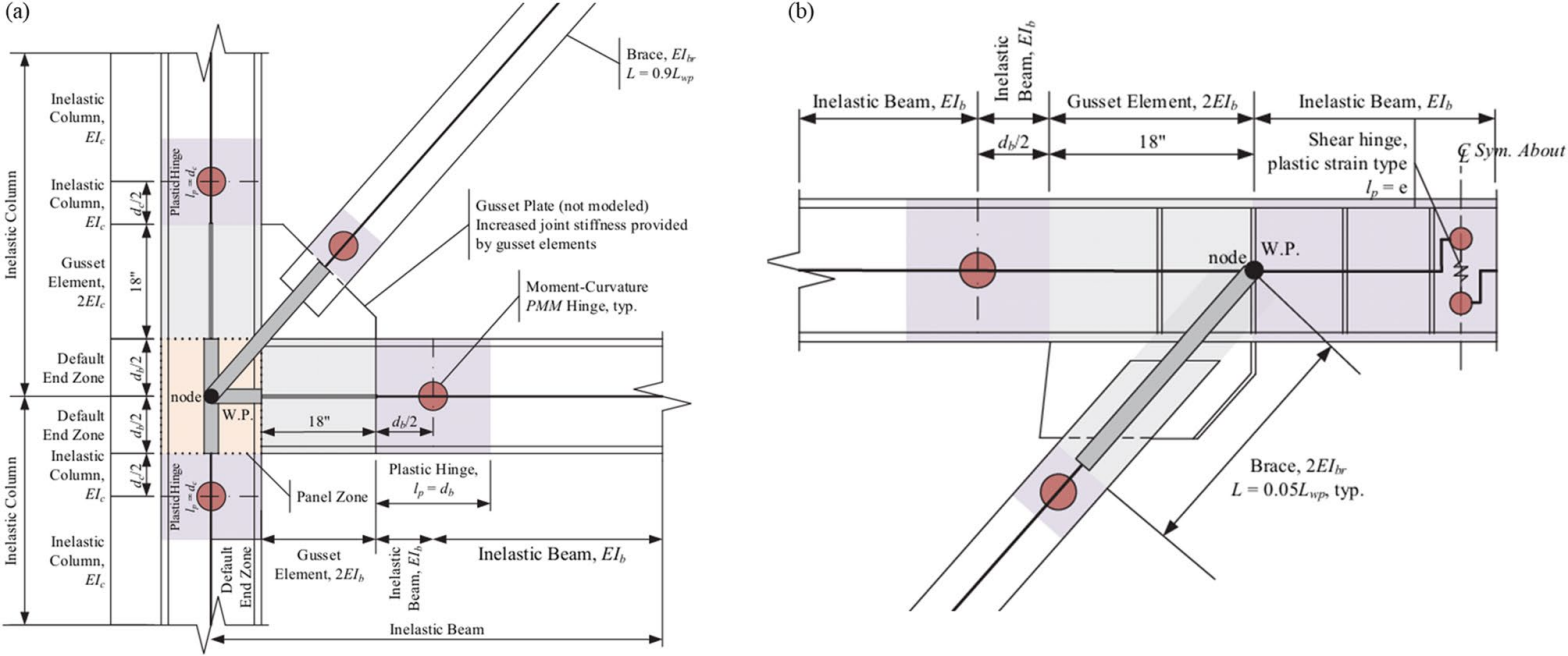
Modelling of EBF beam–column–brace connection and link region.

The modelling of beam–column joints, brace connections, panel zones, stiffeners, and link regions followed the configuration illustrated in Fig. 4^27^. Gusset plates, panel-zone deformation, and link stiffeners were not explicitly modelled as shell elements; instead, their effects were incorporated through equivalent joint stiffness modifiers, ensuring realistic joint flexibility while maintaining computational efficiency. Panel-zone behaviour was calibrated to prevent excessive joint shear deformation and to ensure that inelastic action was concentrated primarily in the designated energy-dissipating components.

Structural steel was modelled using a bilinear elastoplastic constitutive law, exhibiting linear elastic behaviour up to the yield stress followed by post-yield response with limited strain hardening, as implemented in ETABS. Nonlinear behaviour was represented through concentrated plastic hinge formulations. Moment–rotation hinges were assigned at critical beam and column ends to capture flexural yielding, while shear–rotation hinges were assigned over the full length of the EBF link elements to represent shear-dominated inelastic behaviour. The hinge backbone characteristics were defined in accordance with FEMA 356^28^-based provisions, incorporating elastic, yielding, post-yield, and strength degradation stages associated with Immediate Occupancy (IO), Life Safety (LS), and Collapse Prevention (CP) performance levels. The cyclic hysteretic response, including stiffness degradation, strength deterioration, and energy dissipation under load reversals, was implicitly captured through the built-in FEMA hinge formulations available in ETABS, ensuring that inelastic deformation was concentrated within the designated shear link region in accordance with EBF design philosophy.

Special attention was given to the modelling of shear links, which form the primary source of hysteretic energy dissipation in EBF systems. The link region was explicitly defined over its full length, and a nonlinear shear hinge was assigned over the entire link length, ensuring uniform distribution of inelastic shear deformation. The beam segments outside the link region were intentionally designed and modelled to remain elastic throughout the analysis, thereby enforcing the desired link-controlled inelastic mechanism and preventing unintended plastic hinging in beams or columns. All structural members were initially designed using the code-based FBD approach. To enable a clear and unbiased comparison between FBD and DDBD, the same set of member cross sections was intentionally retained for both design frameworks. This modelling strategy eliminates the influence of section-size variability and ensures that any observed differences in seismic response arise solely from the underlying design philosophy rather than changes in member dimensions. Accordingly, the cross-sectional dimensions adopted for beams, columns, braces, and links are reported in Table 5.

The selected sections satisfy strength, stiffness, and drift requirements for all building heights and reflect realistic Indian steel construction practice. ISHB sections were adopted for columns to maintain practical fabrication limits and to avoid built-up sections, even in the 9- and 12-storey frames. Strong-column–weak-beam behaviour was ensured by selecting column sections with higher flexural capacity than the connected beams, thereby preventing column yielding prior to link yielding. Braces were modelled using RHS sections to satisfy axial strength and slenderness limits and to ensure stable cyclic tension–compression behaviour. A single ISMB 400 section was adopted for shear links across all building heights to ensure consistent shear-dominated link behaviour.

**Table 5:** Adopted cross-sectional sizes for beams, columns, braces, and shear links used in numerical modelling.

Storey Frame	Beam Section	Column Section (ISHB)	Brace Section (RHS)	Link Section
3-Storey	ISMB350	ISHB300	RHS 100 × 100 × 6	ISMB400
6-Storey	ISMB400	ISHB350	RHS 150 × 100 × 8	ISMB400
9-Storey	ISMB450	ISHB450	RHS 200 × 100 × 8	ISMB400
12-Storey	ISMB500	ISHB500	RHS 200 × 200 × 10	ISMB400

All steel members were assigned a modulus of elasticity of 200 GPa and a nominal yield strength of 345 MPa. Expected material properties, including overstrength and strain-hardening effects, were incorporated in accordance with IS 18168:2023 ^6^, ensuring realistic representation of post-yield behaviour. Nonlinear behaviour was modelled using user-defined hinge properties calibrated in accordance with ASCE 41 − 17 ^29^, rather than default ETABS hinges. Plastic hinges were assigned at beam and column ends, shear hinges were assigned within the link region, and axial tension–compression hinges were assigned to braces using multilinear backbone curves. The moment–rotation and shear–rotation relationships were defined to reflect stable hysteretic behaviour, and acceptance criteria corresponding to Immediate Occupancy (IO), Life Safety (LS), and Collapse Prevention (CP) were explicitly defined based on ASCE 41-17^29^ recommendations. Overstrength effects were implicitly captured through the use of expected material properties and capacity-based section selection, ensuring that yielding occurred preferentially in the shear links rather than in primary gravity-load-carrying elements.

### Nonlinear seismic analysis framework

Nonlinear static (pushover) and nonlinear dynamic (time-history) analyses were performed to comprehensively evaluate the seismic performance of the 3-, 6-, 9- and 12-storey EBF frames. First, pushover analysis was conducted to obtain the global capacity curve of each frame using a displacement-controlled load pattern proportional to the fundamental mode. The pushover curve was converted into a capacity spectrum to determine the performance point^30^, and hinge formation was evaluated at the ultimate point to assess inelastic mechanisms and link yielding progression. Higher-mode pushover patterns were not included for the 9- and 12-storey buildings because (i) both frames are symmetric and regular in plan and elevation, (ii) their lateral response is dominated by the first mode due to stiffness concentration in the braced bays, and (iii) several prior studies on EBFs report minimal contribution from higher modes in systems with short link lengths.

Nonlinear time-history analysis (NLTHA) was then performed using 16 ground motions selected from the PEER database, covering a wide range of magnitudes, site classes, epicentral distances, and frequency characteristics (Table 6).

**Table 6:** Metadata of selected ground-motion records used in the analysis.

ID	RSN	Event/station	Epicentral distance (km)	Magnitude (Mw)	VS₃₀ (m/s)
1	754	Loma Prieta / Coyote Lake	30.89	6.93	295.01
2	1100	Kobe, Japan / Abeno	46.79	6.90	256.00
3	1186	Chi-Chi, Taiwan / CHY014	66.19	7.62	347.63
4	1628	St. Elias, Alaska / Icy Bay	74.83	7.54	306.57
5	850	Landers / Desert Hot Springs	27.32	7.28	359.98
6	1602	Duzce, Turkey / Bolu	41.27	7.14	293.57
7	5827	El Mayor–Cucapah	18.82	7.20	242.05
8	20	Northern Calif-03	30.49	6.50	219.31
9	730	Spitak, Armenia	36.19	6.77	343.53
10	848	Landers / Coolwater	82.12	7.28	359.82
11	68	San Fernando	39.49	6.61	316.46
12	1101	Kobe, Japan / Amagasaki	38.79	6.90	256.00
13	4849	Chuetsu-oki / Kubikiuka	46.72	6.80	342.74
14	3749	Cape Mendocino / Fortuna Fire	30.04	7.01	355.18
15	1185	Kocaeli, Turkey / Duzce	38.22	7.51	281.86
16	5823	El Mayor–Cucapah	20.63	7.20	242.05

To ensure consistency across the suite, each record was normalised following FEMA 356/P695 (2009), Fema P-58 (2018)^31,32^ guidelines, using the PGA–PGV–spectral-ordinate–based scaling approach also adopted in Rc et al.,2025^33^ and Vyas et al. 2025^34^ For each record, PGA, PGV, and the maximum spectral acceleration in the effective period range were computed, and a normalisation factor was obtained (Table 7). The normalisation reduced dispersion in ground-motion intensity and ensured spectral compatibility. The recorded PGAs ranged from 0.10 to 0.81 g (avg. 0.30 g), while the normalised suite ranged from 0.16 to 0.52 g (avg. 0.31 g); similarly, PGV varied from 20.55 to 65.88 cm/s before normalisation and 33.16–46.42 cm/s after normalisation, ensuring a more consistent velocity content. These normalised accelerograms were then used in NLTHA to extract peak interstorey drift ratios, link plastic rotations, brace forces, residual displacements, and overall structural deformation patterns.

**Fig. 5:**
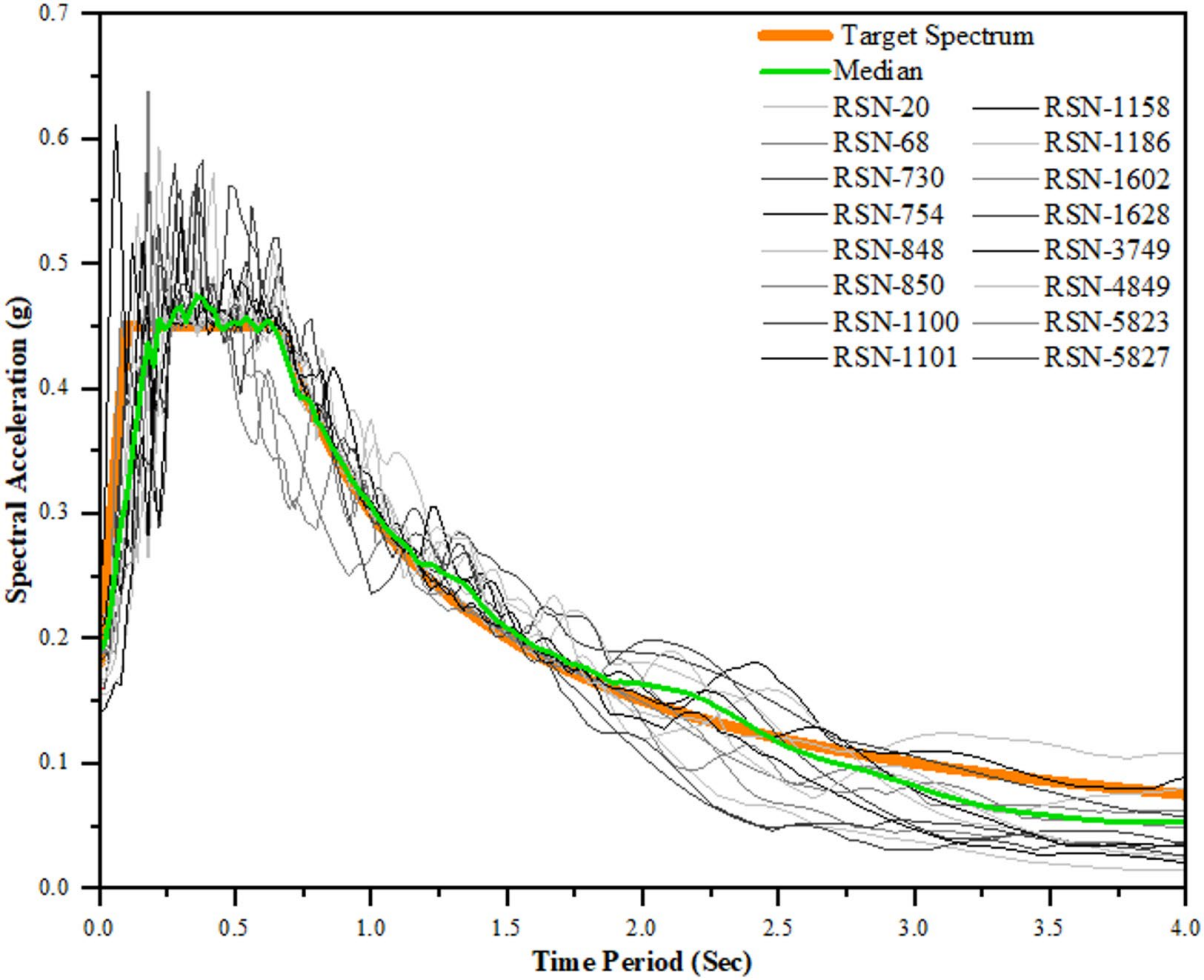
Recorded and normalized response spectra of the selected ground motions compared with the target and median spectra.

The normalisation reduced dispersion in ground-motion intensity and ensured spectral compatibility. Figure 5 illustrates the response spectra of the 16 selected records before and after normalization, plotted together with the target design spectrum and the suite median. The unscaled records show considerable variability in the short-period range (0.1–1.0 s), whereas the normalized suite exhibits substantially reduced scatter and a closer match with the target spectrum. The median spectrum aligns well with the target spectrum across the effective period range of the buildings, confirming that the applied PGA–PGV–spectral-ordinate–based normalization achieved both intensity consistency and spectral compatibility essential for unbiased NLTHA results.

**Table 7:** Peak ground-motion parameters (PGA, PGV) and corresponding normalisation factors computed as per FEMA P695 for developing a spectrally compatible suite of ground motions.

ID	PGA (H₁)	PGA (H₂)	PGV (H₁)	PGV (H₂)	PGV_max_	PGA_max_	Normalised factor	Normalised PGV_max_	Normalised PGA_max_
1	0.16	0.18	13.42	22.72	22.72	0.18	1.87	42.53	0.33
2	0.22	0.23	21.25	24.78	24.78	0.23	1.42	35.30	0.33
3	0.26	0.10	23.05	11.43	23.05	0.26	2.01	46.42	0.52
4	0.10	0.10	19.97	20.55	20.55	0.10	1.61	33.16	0.16
5	0.17	0.15	19.46	20.88	20.88	0.17	1.62	33.86	0.28
6	0.74	0.81	55.93	65.88	65.88	0.81	0.54	35.48	0.44
7	0.54	0.41	61.55	43.54	61.55	0.54	0.63	38.87	0.34
8	0.16	0.20	36.07	26.21	36.07	0.20	1.06	38.35	0.21
9	0.20	0.17	28.36	14.98	28.36	0.20	1.59	44.98	0.32
10	0.28	0.42	27.62	43.42	43.42	0.42	0.94	40.99	0.40
11	0.22	0.19	21.72	16.94	21.72	0.22	1.70	37.01	0.37
12	0.28	0.33	33.57	44.83	44.83	0.33	0.84	37.78	0.28
13	0.21	0.25	38.63	43.89	43.89	0.25	0.79	34.84	0.20
14	0.33	0.28	33.91	38.05	38.05	0.33	0.91	34.63	0.30
15	0.31	0.36	58.87	55.66	58.87	0.36	0.57	33.62	0.21
16	0.25	0.20	38.34	33.99	38.34	0.25	0.91	34.72	0.23

Incremental Dynamic Analysis (IDA) was subsequently conducted to capture structural behaviour over a wide intensity range, following the classical methodology proposed by Luco and Cornell 2007^35^; Verki and Aval 2020^36^. The normalised accelerograms were scaled progressively until collapse or numerical instability, and IDA curves were developed using spectral acceleration at the effective period (Sa(T₁,5%))^37^ as the intensity measure and peak interstorey drift as the damage measure.

## Results

### Pushover response

**Fig. 6:**
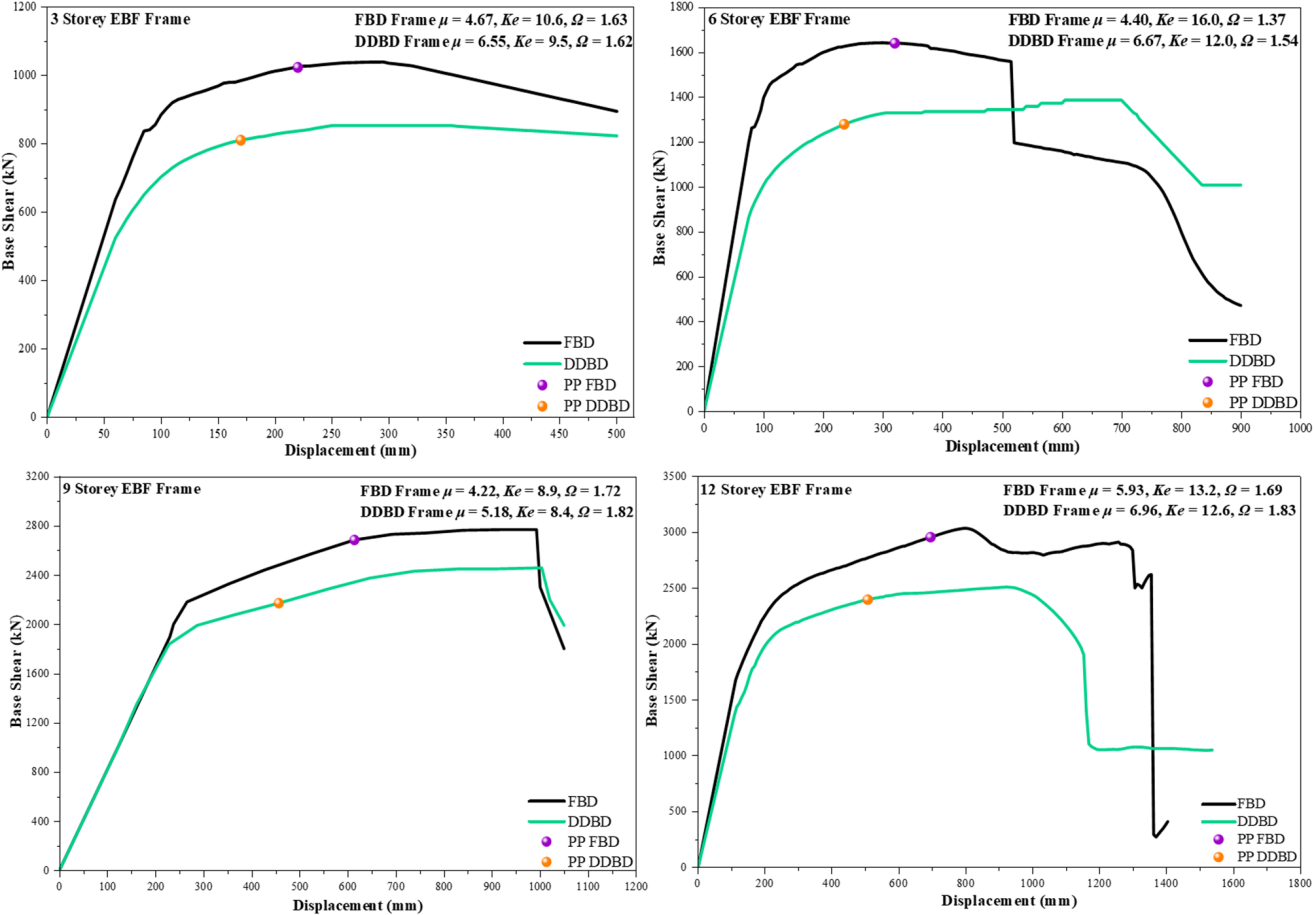
**(a–d)**. Pushover curves for the 3-, 6-, 9-, and 12-storey EBF buildings designed using FBD and DDBD.

Pushover analysis results for the 3-, 6-, 9-, and 12-storey EBF frames are presented in Fig. 6**(a–d)** in terms of base shear versus roof displacement, providing insight into the strength, stiffness, ductility, and post-yield behaviour of frames designed using FBD and DDBD approaches. For all building heights, the FBD frames exhibit higher initial stiffness and higher yield base shear, with yield strengths exceeding those of the corresponding DDBD frames by approximately 10–18%, indicating a force-oriented design emphasis. However, this higher strength is accompanied by earlier stiffness degradation and pronounced post-peak softening, particularly in the 6-, 9-, and 12-storey frames, where a rapid drop in base shear is observed beyond the peak response. In contrast, the DDBD frames demonstrate a smoother transition from elastic to inelastic response, followed by a stable post-yield plateau that is sustained over a significantly larger displacement range.

The ultimate displacement capacity ($\:{{\Delta\:}}_{max}$) of the DDBD frames is consistently higher than that of the FBD frames, resulting in ductility ratios ($\:\mu\:$), that are approximately 15–30% greater, depending on building height. This enhanced ductility reflects the deliberate concentration of inelastic deformation within the shear links in DDBD-designed frames, which limits damage in primary structural members and delays global strength deterioration. Although the initial stiffness ($\:{K}_{e}$*)* of FBD frames is higher leading to reduced displacements at low demand levels it also promotes stiffness-controlled response and localised hinge formation, which contributes to abrupt post-peak strength loss in taller frames. Conversely, the moderated stiffness of DDBD frames enables controlled deformation, preventing sudden strength degradation and allowing more uniform energy dissipation along the height.

### Plastic hinge distribution and inelastic behaviour at the ultimate load

**Fig. 7:**
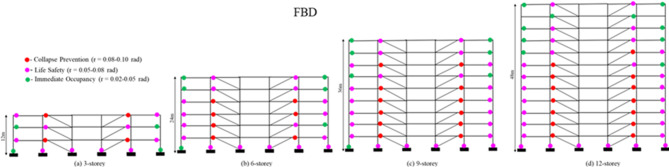
**(a-d).** Plastic hinge distribution at the performance point for the 3-, 6-, 9-, and 12-storey FBD frames.

**Fig. 8:**
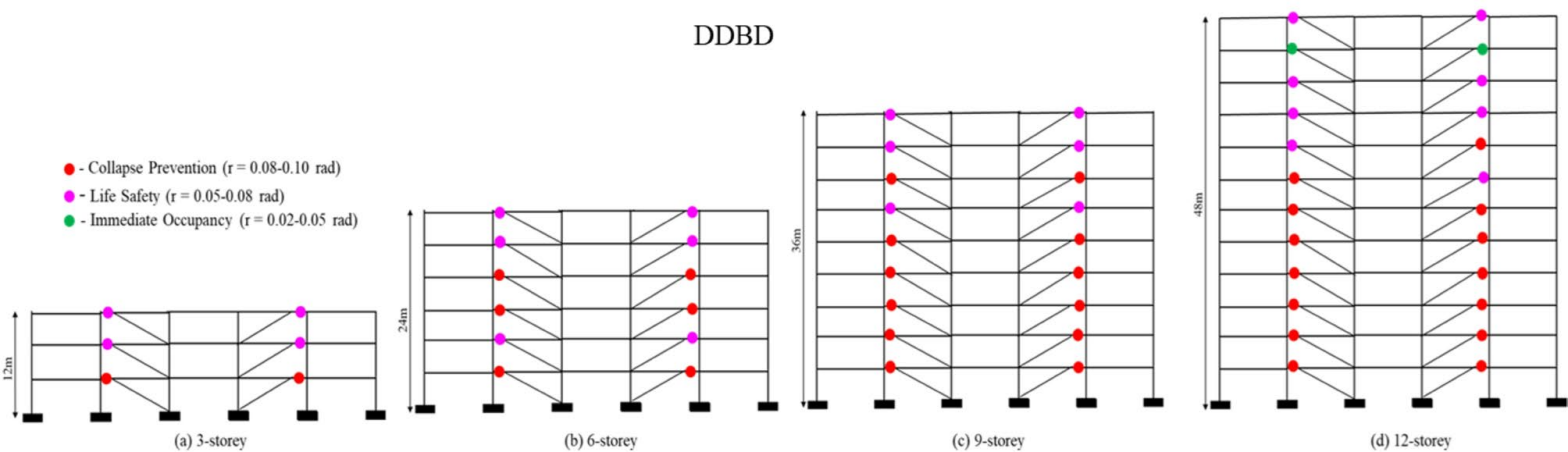
**(a-d)**. Plastic hinge distribution at the performance point for the 3-, 6-, 9-, and 12-storey DDBD frames.

The plastic hinge patterns shown in Figs. 7 and 8 correspond to the structural response at a target inter-storey drift ratio of 4%, which represents the ultimate (near-collapse) deformation state of the frames. The distribution of plastic rotations at this drift level reveals clear differences in how the FBD and DDBD frames mobilize their inelastic mechanisms along the building height. For all building configurations, plastic rotations in the shear links are not uniformly distributed. Upper-storey links generally exhibit very small rotations, indicating limited participation in energy dissipation due to significantly lower storey shear demands at higher elevations. In contrast, the lower and second-to-middle storeys consistently develop much larger plastic rotations, demonstrating that these levels dissipate the majority of the input seismic energy.

In the FBD-designed frames, the concentration of inelasticity becomes increasingly irregular with increasing building height. In the 9- and 12-storey frames, a large proportion of links in the lower and second-to-middle storeys reach Collapse Prevention (CP)-level deformation, while several adjacent storeys simultaneously experience Life Safety (LS)-level demands, indicating localized overstressing and a tendency toward soft-storey-type behaviour. At the 4% drift level, approximately 40–60% of the links in the critical lower storeys of the FBD frames reach CP-level rotations, reflecting poor control over deformation distribution. This behaviour can be attributed to the force-based design philosophy, which prioritizes strength and initial stiffness but does not explicitly regulate inter-storey drift, leading to abrupt stiffness degradation and concentration of plastic demand.

The overstrength factor (Ω) further highlights this behavioural distinction. While FBD frames develop relatively higher overstrength, particularly in taller configurations, this reserve capacity is often mobilised in a concentrated manner, resulting in undesirable hinge clustering and rapid softening. DDBD frames, on the other hand, exhibit moderate and more uniformly distributed overstrength, which supports stable inelastic response without excessive strength loss. The performance point (PP) responses reinforce these observations: for the 6-, 9-, and 12-storey buildings, the PP drift demands of DDBD frames are reduced by approximately 20–40% compared to FBD frames, indicating superior displacement control and improved damage limitation.

Consistent with these observations, the DDBD-designed frames exhibit a more stable and predictable inelastic mechanism at the same drift level. Although the lower and second-to-middle storeys still attract the largest plastic rotations, the demands are more uniformly distributed along the height, and the number of links reaching CP-level deformation is significantly reduced. At the 4% drift state, only about 15–25% of the links in DDBD frames approach CP-level rotations, with the majority remaining within LS limits. Moreover, inelastic action in DDBD frames remains largely confined to the shear links, with no significant plastic hinging observed in beams or columns, confirming the effectiveness of displacement-based design in enforcing a link-controlled mechanism. This uniform distribution of plastic demand results in improved drift control, reduced risk of localized failure, and a safer and more reliable energy-dissipation pattern compared to FBD frames.

### IDA drift demand and variability

Incremental Dynamic Analysis (IDA) was employed to investigate the evolution of drift demand and its record-to-record variability under progressively increasing seismic intensity. In IDA, each ground motion record is incrementally scaled and applied repeatedly until structural collapse is observed, as originally proposed by Vamvatsikos and Cornell (2004)^35^. Unlike nonlinear static pushover analysis, IDA directly applies real earthquake time histories and therefore provides a more realistic representation of nonlinear dynamic response, particularly for capturing collapse-level behaviour. In the present study, a suite of 16 ground motions was considered, and each record was scaled over a wide range of intensity levels to ensure full coverage of elastic, inelastic, and near-collapse response regimes. Intensity levels were increased incrementally to generate a dense set of response data, enabling reliable estimation of drift demand and variability.

**Fig. 9:**
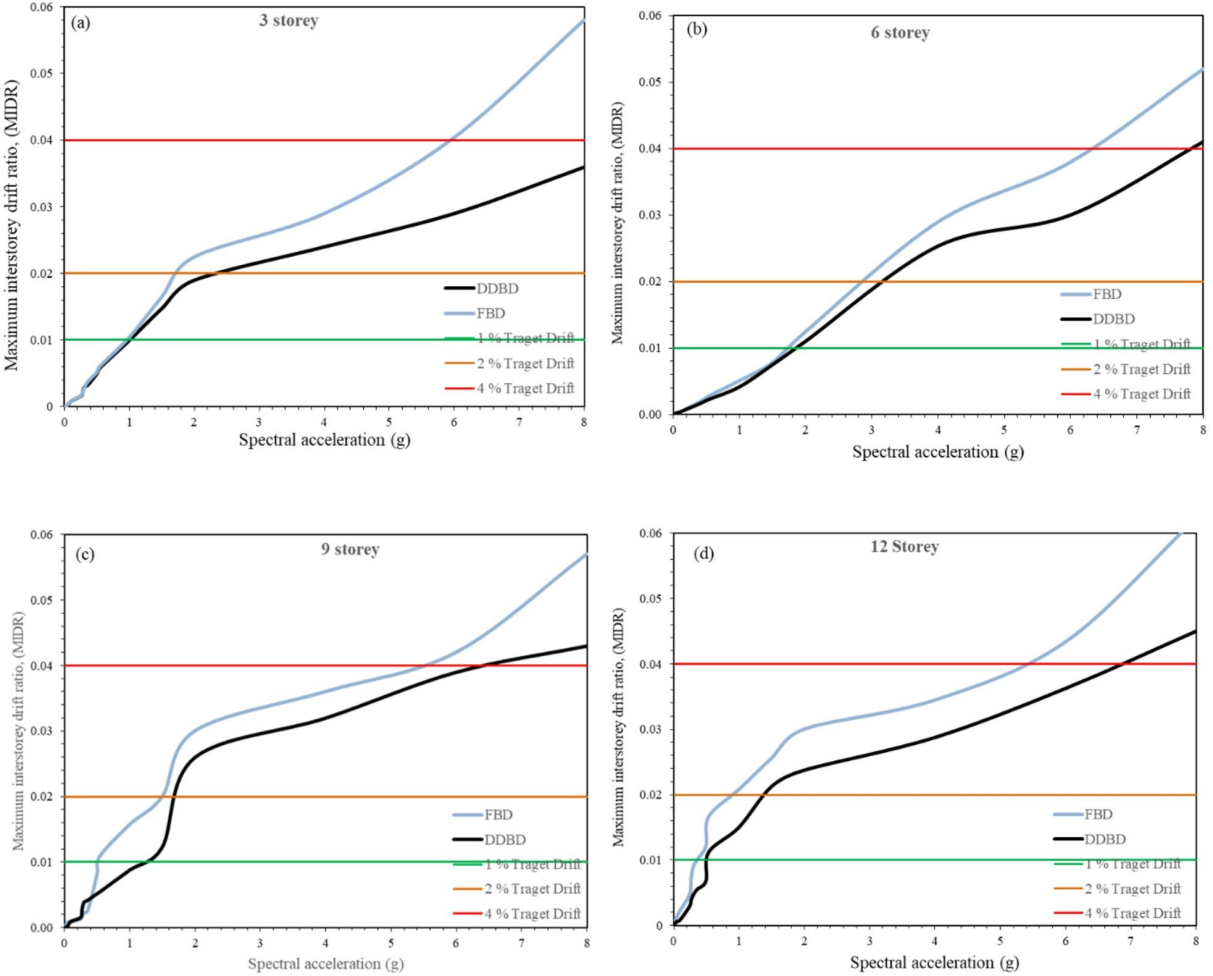
**(a-d)**. IDA curves (Sa vs. MIDR) for 3-, 6-, 9-, and 12-storey steel frames designed using FBD and DDBD, with 1%, 2% and 4% target drift limits.

The median IDA curves for the 3-, 6-, 9-, and 12-storey frames, expressed in terms of maximum inter-storey drift ratio versus spectral acceleration (MIDR- Sa), are presented in Fig. 9**(a–d)**. These graphs demonstrate definite and systematic differences in deformation evolution patterns FBD and DDBD systems. In the case of the 3-story structure, it can be observed that both design methods begin to follow broadly similar courses at first; however, FBD’s curve tends to accelerate significantly just after 1% MIDR, depicting an early entry into strongly nonlinear dynamics, while in DDBD, it tends to remain at a shallower slope over a larger deformation span. In the case of the 6-story structure, it can be found that the departure in curves happens at lower levels of drift, with FBD’s curve strongly accelerating in the interval of 1% to 4% drift, while in DDBD, it tends to remain more gradual, touching the region of 2% drift at a higher SA mark.

The difference is more extreme for 9-and 12-story buildings, as the FBD curves reflect a rapid drift demand escalation, which has a clear upward convex portion shortly after 1.2% MIDR. By contrast, the DDBD curves demonstrate a gradual slope that prolongs before another acceleration phase, which represents a delayed accumulation of inelastic deformation. Generally, for all building heights, the knee, which corresponds to the onset of a significant drift demand increment, is found to lie at lower levels for FBD systems than DDBD systems. Also, the DDBD curves meet both 1% and 2% drift levels at higher Sa, whereas the FBD curves satisfy these levels earlier, which signify basic differences between deformation controls. For higher buildings, it is noted that horizontal extension becomes more pronounced for FBD systems, which reveal a rapid drift development per seismic level increment, while DDBD systems are observed to become more elongated, especially for 9-and 12-story buildings.

**Fig. 10:**
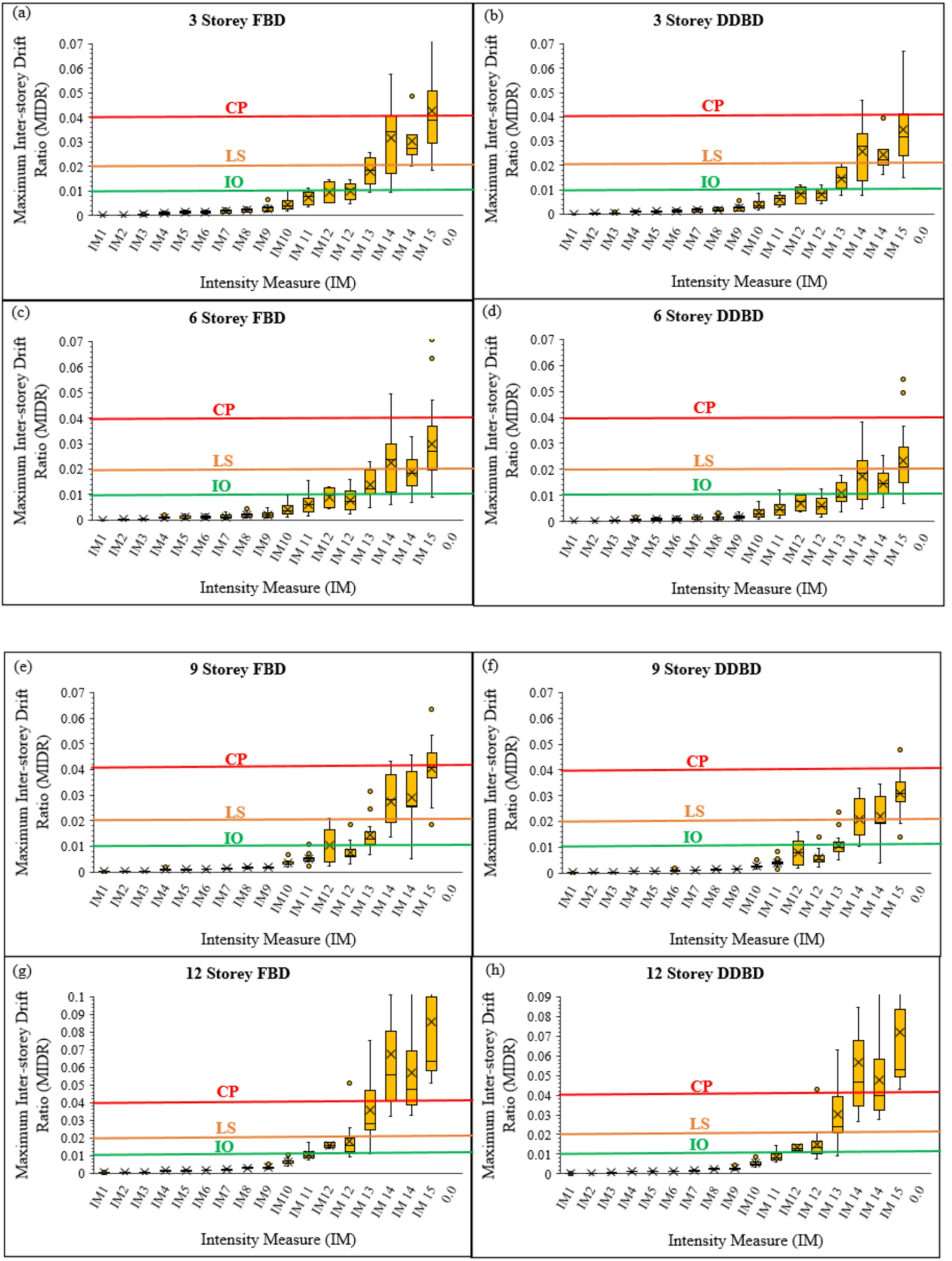
**(a-h).** Maximum inter-storey drift ratio (MIDR) versus intensity measure (IM) for 3-, 6-, 9-, and 12-storey steel frames designed using FBD and DDBD.

To complement the median IDA curves and explicitly quantify record-to-record variability, the drift responses obtained from all scaled analyses were consolidated using MIDR–IM box plots, shown in Fig. 10**(a–h)**. These box plots summarise the drift response from the 16 ground motions, each incrementally scaled across multiple intensity levels, resulting in an extensive dataset essential for capturing the full nonlinear behaviour of the frames. The box-type representation enables simultaneous visualisation of the median, dispersion, and extreme values, making it particularly suitable for performance-based seismic assessment where uncertainty plays a critical role. Across all building heights, MIDR increases progressively with increasing seismic intensity; however, clear behavioural differences emerge between FBD and DDBD designs.

In the case of 3- and 6-story frames, both design methods remain bound by fairly small drift ranges at low intensity, while nonlinear behavior related to increased intensity starts to appear. For the 9- and 12-story frames, higher modes push up drift demand, especially in FBD systems, displaying larger medians, more dispersion, and many outliers. Many FBD responses reach or exceed common limits, such as 1–1.5% for Life Safety and 2.5–4% for Collapse Prevention, at higher intensity, and this points to greater vulnerability and poorer control of deformation. On the contrary, the DDBD frames show lower median drift demand and tighter clustering consistently in all intensity levels, thus revealing the underlying effectiveness of preset target displacement, stable energy dissipation, and controlled plastic hinging coming with a displacement-based design.

### Link rotation demand under SLE, DBE, and MCE Hazard Levels

**Fig. 11:**
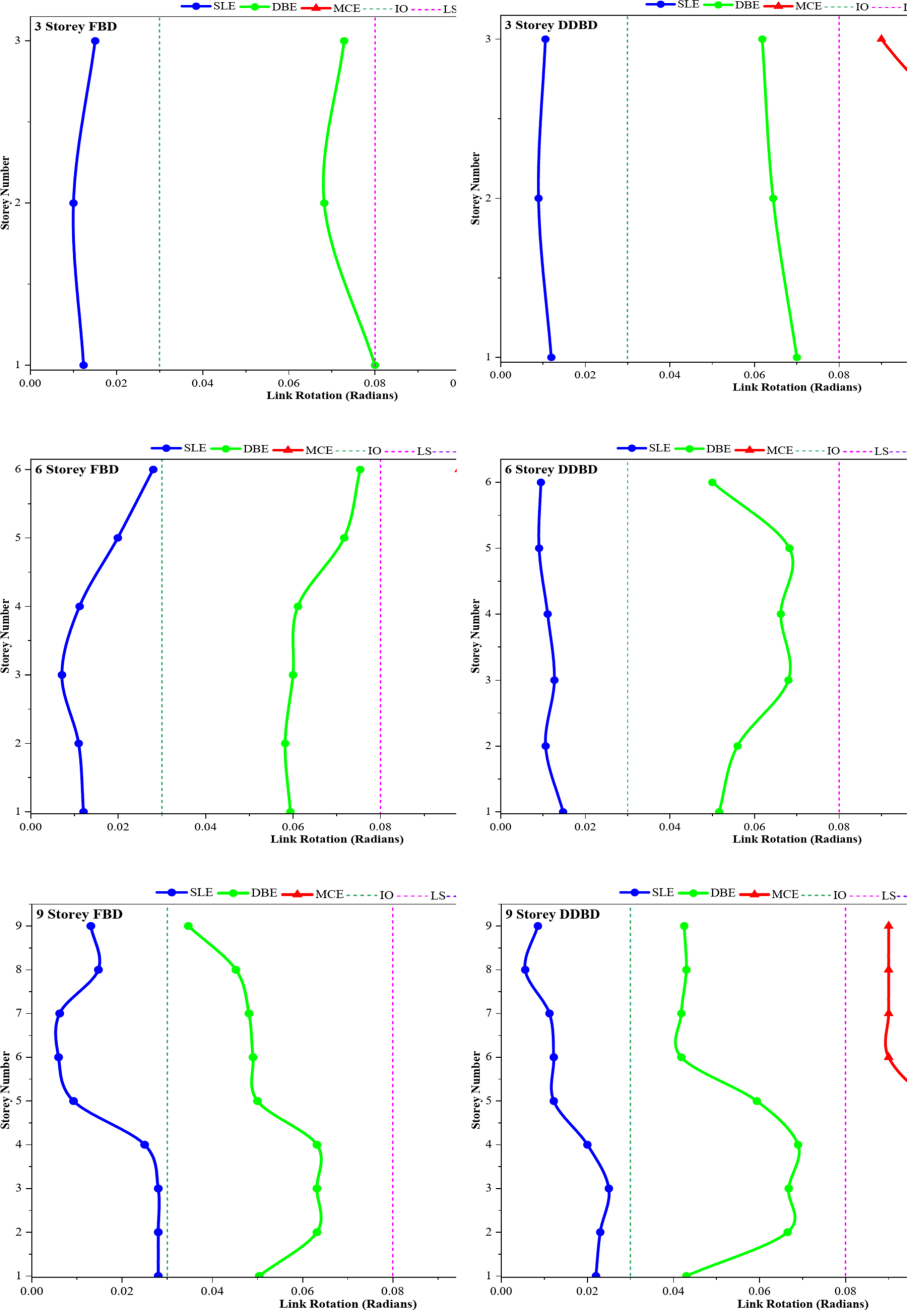

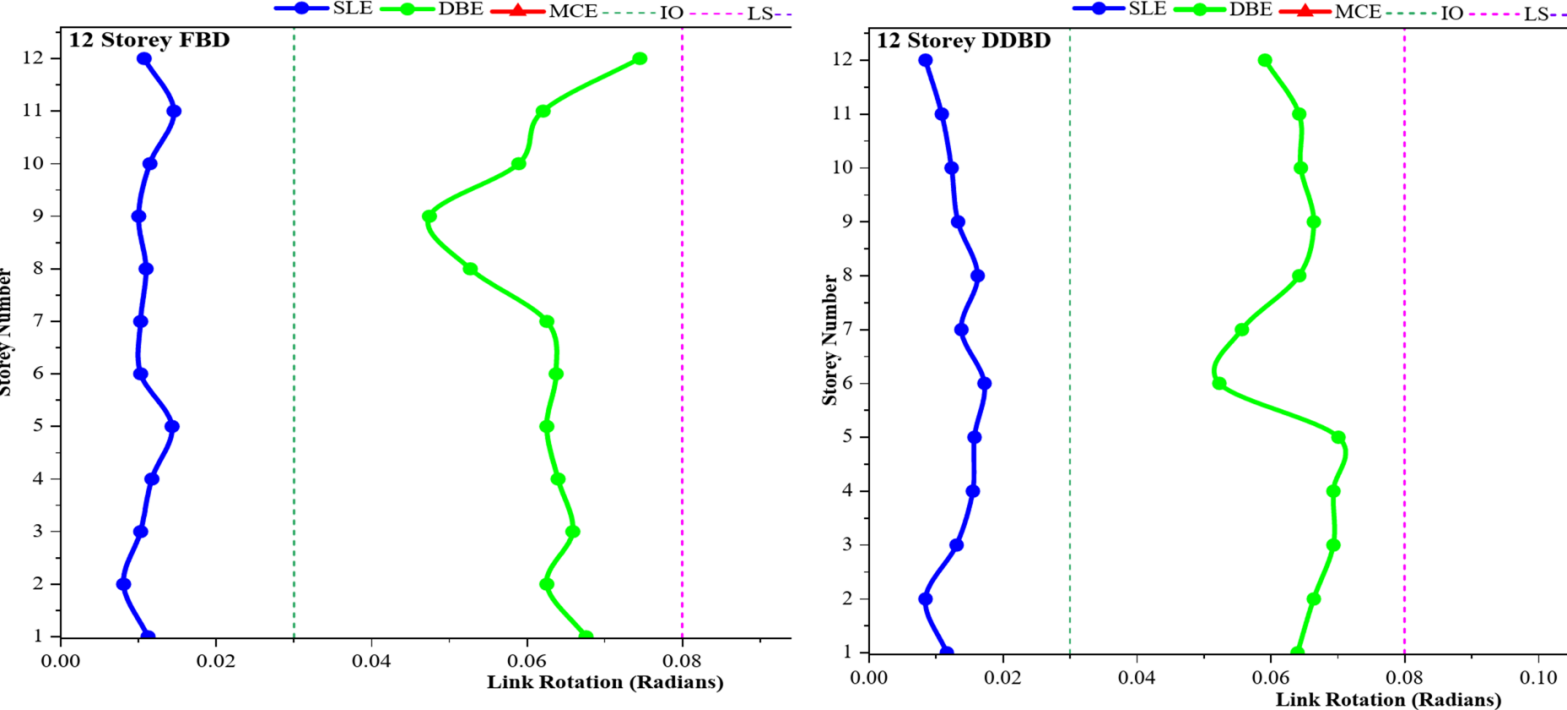
**(a-h).** Distribution of link rotation demands across different storeys for 3-, 6-, 9-, and 12-storey EBF frames that were designed utilizing FBD and DDBD methodologies under seismic hazard levels of SLE, DBE, and MCE.

Link-rotation profiles in Fig. 11**(a–h)** represent the median rotation demands calculated from nonlinear time-history analyses considering all ground motions in the entire spectrum at each hazard level, namely SLE (0.18 g), DBE (0.27 g) and MCE (0.54 g). Use of median values helps to reduce record-to-record variability and provides a stable, representative estimate of the underlying structural response. Independent of building heights, frames designed using FBD demonstrate a uniform higher median link-rotation demands and increased sensitivity to increasing hazard intensity, reflecting the force-controlled nature of the design methodology.

At the SLE, the link rotations are well below the yield rotation threshold, indicating predominantly elastic behavior and satisfactory serviceability performance in both the FBD and the DDBD frames. However, clear distinctions can be observed for higher hazard levels. For DBE excitation, the FBD frames show significant inelastic link rotations, with multiple stories reaching close to the LS deformation range. These trends are notably more pronounced at the MCE level, where median link rotation demands in the 6-, 9-, and 12-story FBD frames frequently reach values in the vicinity of approximately 0.10–0.14 rad. These demands approach or exceed commonly accepted shear-link rotation limits for AISC 341 / ASCE 41-17^39^ (IO ≈ 0.03 rad, LS ≈ 0.08 rad, CP ≈ 0.12 rad), indicating that several stories experience LS to CP level deformation, while some links from these stories exceed the CP threshold and enter a potential failure regime.

In addition to higher absolute rotation demand, the FBD frames exhibit a clear height-dependent concentration of link rotation, indicative of an unfavourable plastic mechanism. For the 6-storey frame, peak rotations tend to localise in the mid-height storeys, whereas in the 9-storey frame the concentration shifts toward the upper storeys. In the 12-storey frame, pronounced rotation spikes are observed across both mid- and upper-storey regions. This spread of plastic demand becomes uneven because changes in stiffness and force-based design push the drift to concentrate. It also acts as an indication of higher risk for soft-storey behaviour when earthquakes are strong.

But frames designed with DDBD show a much more controlled and predictable response across different hazard levels. The median link rotations follow a smooth, almost straight path and are more uniformly distributed along the building height. Even under MCE-level shaking, the median rotation demand for DDBD frames stays within 0.06–0.08 rad, stays within Life Safety, and remains below the CP threshold. Compared to FBD frames, this represents a reduction of roughly 30–50% in median rotation demand at MCE levels, although the exact amount depends upon building height. The lack of abrupt rotation increases at any floor suggests that drift concentration is duly accounted for within these displacement-based designs.

The enhanced performance of DDBD frames is directly linked to the explicit management of target displacement and the deliberate creation of inelastic mechanisms, which fosters a more uniform distribution of plastic demand and limits inelasticity to the specified shear links. This benefit becomes increasingly evident with greater building heights, especially in the 9- and 12-storey frames, where FBD systems show significant rotation concentration while DDBD systems maintain consistent and well-distributed deformation patterns. Overall, the link rotation outcomes indicate that DDBD offers superior deformation control, a lower likelihood of exceeding critical rotation limits, and improved seismic resilience across all hazard levels when assessed against traditional force-based design.

## Conclusions

This paper investigates the seismic performance of eccentrically braced steel frames designed according to the newly developed Indian Standard IS 18168:2023. Two design approaches have been considered in this study, namely conventional FBD and DDBD. EBF buildings of 3, 6, 9, and 12 stories have been considered. Strength, ductility, development of inelastic mechanisms, and behavior at or approaching collapse have been studied by nonlinear static pushover, nonlinear time-history, and incremental dynamic analyses.

1. Eccentrically braced steel frames (EBFs) that are designed in alignment with IS 18168:2023 exhibit stable and dependable seismic performance, with inelastic deformation mainly focused on the specified shear links. For all building heights, both design methods effectively engage the intended energy-dissipation mechanism, confirming the efficacy of the latest Indian seismic regulations for steel structures.
2. Pushover results show that FBD-designed buildings have a yield capacity of 10%–20% higher stiffness and strength but soften more after yielding and are less stable in deformation, while DDBD-designed buildings are 15%–30% more stable in displacement ductility.
3. Nonlinear time-history results also showed that median inter-story drifts for the DDBD system are about 20%–40% less than those from FBD systems, depending on building height. Second, record-to-record variability in drift response is also reduced by about 25%–50% for the DDB systems, which implies that their responses will be more predictable for strong inputs.
4. Incremental Dynamic Analysis (IDA) findings further validate that DDBD frames postpone the initiation of rapid drift escalation by roughly 20–30% regarding intensity measure, especially in 9- and 12-storey structures. FBD frames demonstrate earlier knee points and reduced collapse intensities, while DDBD frames ensure consistent deformation growth across a broader intensity spectrum.
5. Evaluation of link rotation demands at SLE (0.18 g), DBE (0.27 g), and MCE (0.54 g) reveals that FBD frames experience 30–50% higher median link rotations at MCE, with several storeys approaching or exceeding Collapse Prevention limits. In contrast, DDBD frames limit median MCE-level link rotations to within Life Safety limits, achieving an overall reduction of approximately 30–50% in critical rotation demand.
6. The distribution of plastic hinges at near-collapse drift levels suggests that FBD frames tend to develop localized clusters of plasticity, thereby heightening the chances of soft-storey-type mechanisms, particularly in taller structures. In contrast, DDBD frames demonstrate a more even distribution of plastic demand, leading to a decrease of about 40–60% in the number of links experiencing CP-level deformation.
7. Overall, the combined results demonstrate that while IS 18168:2023 provides a robust framework for EBF design, the integration of Direct Displacement-Based Design enhances seismic performance by approximately 20–50% across key response parameters, including drift demand, deformation concentration, and collapse safety.
8. Based on the comprehensive comparison, it is concluded that DDBD-designed EBFs offer a more realistic and performance-oriented representation of nonlinear seismic behaviour, particularly in the moderate-to-strong shaking range, making them a highly effective design approach for steel buildings in seismic regions.

Future studies may extend the present work by incorporating soil–structure interaction and foundation flexibility, which can influence displacement demand and link rotation behaviour in steel EBF systems. The applicability of DDBD-based EBF design may also be explored for irregular and asymmetric buildings, where higher-mode and torsional effects become more pronounced. Experimental or hybrid simulation studies on full-scale or sub-assemblage EBF link components are recommended to further validate the numerical findings under cyclic and near-collapse loading conditions. In addition, the integration of probabilistic performance metrics, such as fragility and loss assessment, would provide deeper insight into the risk-based advantages of DDBD-designed EBFs under region-specific seismic hazard scenarios.

## Data Availability

The data supporting this study’s findings are available from the corresponding author, Bush Rc, upon reasonable request.
